# Shelby Medical College

**Published:** 1858-08

**Authors:** 


### Shelby Medical College.

Since our last issue we have received the announcement of the Shelby Medical College, Nashville, Tennessee, the circular from which was noticed in the last number.

We have already expressed our good wishes for the success of this enterprize; and since we have had an opportunity of seeing the names of the entire Faculty, we have no hesitation in predicting for it in a few years, at least, an equal position with the Medical College previously established in that city. The following gentlemen compose the Faculty: Jno. Frederick May, M. D., of Washington City, D. C. Prof. of the Principles and Operations of Surgery; E. B. Haskins, M. D., of Clarksville, Tennessee, Prof. of the Theory and Practice of Medicine; John P. Ford, M. D., of Nashville, Tenn., Prof. of Obstetrics and Diseases of Women and Children; Thomas L. Maddin, M. D., of Nashville, Tenn., Prof. of Anatomy—Descriptive, Histological and Surgical; John H. Callender, M. D., of Nashville, Tenn., Prof. of Materia Medica and Therapeutics; Richard O. Currey, M. D., of Knoxville, Tenn., Prof. of Medical Chemistry and Medical Jurisprudence; Daniel F. Wright, M. D., of Memphis, Tenn., Prof. of Physiology and Pathology; R. C. K. Martin, M. D., of Nashville, Tenn., Prof. of Clinical Medicine; H. M. Compton, M. D., Demonstrator of Anatomy.

